# Prefrontal EEG slowing, synchronization, and ERP peak latency in association with predementia stages of Alzheimer’s disease

**DOI:** 10.3389/fnagi.2023.1131857

**Published:** 2023-03-23

**Authors:** Jungmi Choi, Boncho Ku, Dieu Ni Thi Doan, Junwoo Park, Wonseok Cha, Jaeuk U. Kim, Kun Ho Lee

**Affiliations:** ^1^Human Anti-Aging Standards Research Institute, Uiryeong-gun, Republic of Korea; ^2^Digital Health Research Division, Korea Institute of Oriental Medicine, Daejeon, Republic of Korea; ^3^School of Korean Convergence Medical Science, University of Science and Technology, Daejeon, Republic of Korea; ^4^Gwangju Alzheimer’s Disease and Related Dementias Cohort Research Center, Chosun University, Gwangju, Republic of Korea; ^5^Department of Biomedical Science, Chosun University, Gwangju, Republic of Korea; ^6^Dementia Research Group, Korea Brain Research Institute, Daegu, Republic of Korea

**Keywords:** electroencephalography (EEG), event-related potential (ERP), Alzheimer’s disease (AD), mild cognitive impairment (MCI), amyloid deposition, neurodegeneration

## Abstract

**Background:**

Early screening of elderly individuals who are at risk of dementia allows timely medical interventions to prevent disease progression. The portable and low-cost electroencephalography (EEG) technique has the potential to serve it.

**Objective:**

We examined prefrontal EEG and event-related potential (ERP) variables in association with the predementia stages of Alzheimer’s disease (AD).

**Methods:**

One hundred elderly individuals were recruited from the GARD cohort. The participants were classified into four groups according to their amyloid beta deposition (A+ or A−) and neurodegeneration status (N+ or N−): cognitively normal (CN; A−N−, *n* = 27), asymptomatic AD (aAD; A + N−, *n* = 15), mild cognitive impairment (MCI) with AD pathology (pAD; A+N+, *n* = 16), and MCI with non-AD pathology (MCI(−); A−N+, *n* = 42). Prefrontal resting-state eyes-closed EEG measurements were recorded for five minutes and auditory ERP measurements were recorded for 8 min. Three variables of median frequency (MDF), spectrum triangular index (STI), and positive-peak latency (PPL) were employed to reflect EEG slowing, temporal synchrony, and ERP latency, respectively.

**Results:**

Decreasing prefrontal MDF and increasing PPL were observed in the MCI with AD pathology. Interestingly, after controlling for age, sex, and education, we found a significant negative association between MDF and the aAD and pAD stages with an odds ratio (OR) of 0.58. Similarly, PPL exhibited a significant positive association with these AD stages with an OR of 2.36. Additionally, compared with the MCI(-) group, significant negative associations were demonstrated by the aAD group with STI and those in the pAD group with MDF with ORs of 0.30 and 0.42, respectively.

**Conclusion:**

Slow intrinsic EEG oscillation is associated with MCI due to AD, and a delayed ERP peak latency is likely associated with general cognitive impairment. MCI individuals without AD pathology exhibited better cortical temporal synchronization and faster EEG oscillations than those with aAD or pAD.

**Significance:**

The EEG/ERP variables obtained from prefrontal EEG techniques are associated with early cognitive impairment due to AD and non-AD pathology. This result suggests that prefrontal EEG/ERP metrics may serve as useful indicators to screen elderly individuals’ early stages on the AD continuum as well as overall cognitive impairment.

## 1. Introduction

Dementia is a group of neurodegenerative disorders that affect human cognition and behaviors, such as decline in memory, impairment in reasoning and language ability, and changes in personality and behavior (McKhann et al., 2011). Alzheimer’s disease (AD) is the main cause of dementia, accounting for approximately 60–80% of cases (Alzheimer’s Association, 2022). Due to the aging of our society, the prevalence of AD dementia has been increasing continuously, representing a major issue for public welfare (Alzheimer’s Association, 2022). The progressive accumulation of amyloid-beta (Aβ) plaques and neurofibrillary tau tangles represent bio-pathological structures in the AD brain (Sperling et al., 2011; Jack et al., 2018). Notably, the pathophysiological process of AD may begin many years before the onset of clinical symptoms (Bateman et al., 2012; Villemagne et al., 2013). At the preclinical stage, cognitive abilities are not yet impaired, but evidence of cortical Aβ deposition may already be present; this is considered the most upstream process in the pathological cascade of AD (Jack, et al., 2013). By the time dementia clinically manifests, brain damage due to synaptic loss and neuronal dysfunction has become irreversible (Kumar et al., 2022). To date, there is no cure for AD or any type of dementia (Kumar et al., 2022). Therefore, detecting cognitive impairment due to AD at its early stages is an urgent prerequisite for therapeutic treatment to slow or halt disease progression before major brain damage occurs (Sperling et al., 2011). The key to this success is the discovery of reliable biomarkers that can distinguish preclinical and prodromal AD from cognitively normal (CN) aging.

Clinically, AD dementia is detected by comprehensive evaluation procedures, which may include history assessment, neurological examinations, neuropsychological tests, and brain imaging (Bon et al., 2011). In addition, cerebrospinal fluid (CSF) Aβ and tau have been considered as core biomarkers of AD in clinical diagnosis and research framework (Blennow, 2017); the decrease of Aβ peptide (i.e., Aβ_42/40_) and the increase of tau protein (i.e., threonine P-tau_181_) are commonly reported as potential CSF indicators of AD dementia (Andreasen et al., 1999; Ashton et al., 2022). Recently, research attempt to detect AD dementia using fluid Aβ and tau obtained from plasma or serum as alternative approaches (Lee et al., 2019). Similar to CSF, plasma Aβ and tau protein concentrations have been found in AD with similar trends. However, these findings suffer from inconsistent results across cognitive stages and low diagnostic accuracy (Song et al., 2011; Feinkohl et al., 2020). Other imaging techniques such as magnetic resonance imaging (MRI) and/or positron emission tomography (PET) can be alternatively used to provide practical information on the biological construct of AD pathology as well as the structure and functions of the brain. Nevertheless, they suffer from several limitations that restrict accessibility, such as being costly, invasive (e.g., radiotracers are injected to measure the levels of Aβ in the human brain on PET scan), relying on specializer’s expertise, and time-consuming testing procedures (Delso et al., 2015). The need for a simpler and more accessible brain imaging method has led to an increase in the use of electroencephalography (EEG) to screen AD stages.

Electroencephalography is a method to record the electrical activities of cortical pyramidal neurons in the brain (Monllor et al., 2021). When there is a large number of neurons fired synchronously, sufficient postsynaptic potentials can be produced and thus detectable from the scalp of the brain. The analysis of EEG data therefore can reflect the normal brain functions and abnormal neural activities such as in AD. This scalp EEG method is not only non-invasive, relatively safe, and fast but also inexpensive, widely available, and allows repeated measurements on a vast number of high-risk elderly individuals (Tsolaki et al., 2014). Additionally, variables obtained from EEG variants [i.e., event-related potential (ERP)] can overcome the cultural and educational limitations that may otherwise restrict the feasibility of conducting neuropsychological tests (Polich and Corey-Bloom, 2005). ERPs are very small voltages occurred on the EEG signal as responses to specific events or stimuli (Blackwood and Muir, 1990). These voltage peaks on the ERP signals are time-locked to specific events that reflect the sensation, perception, and cognition of the human brain. ERPs therefore can provide physiological and pathological components related to human cognitive ability.

Numerous studies have validated the feasibility and reliability of resting-state EEG and ERP in relation to cognitive impairment due to AD (Cassani et al., 2018; Paitel et al., 2021; Tarawneh et al., 2021). Notably, EEG spectral measures, synchronization patterns, and ERP latencies have served as potential indicators to screen individuals with mild cognitive impairment (MCI) and AD dementia (Garcés et al., 2013; Paitel et al., 2021; Sedghizadeh et al., 2022). EEG spectral analysis captures the fluctuations of the electrical brain signals in the form of frequency with respect to their amplitude (Monllor et al., 2021). In EEG spectral measures, the frequency components (e.g., peak frequency) reflect the speed of neural oscillation and power density features (e.g., spectrum triangular index) reveal the cortical neural synchronization which are often seen in neurodegenerative patients (Garcés et al., 2013; Paitel et al., 2021; Sedghizadeh et al., 2022). While EEG avoids any form of stimuli, ERP takes the reflection of the brain into account when interacting with a particular stimulus (Blackwood and Muir, 1990). A delayed response on ERP signals demonstrates a deterioration in the cognitive ability to process and recognize the stimuli. Consequently, a slowing of oscillatory brain activity, a reduction in regional brain synchronization and complexity, and a prolonged peak latency are often exhibited in individuals with MCI and AD dementia compared to CN persons (Garcés et al., 2013; Paitel et al., 2021; Sedghizadeh et al., 2022).

As documented in previous studies, the slowing of brain oscillations has been captured by EEG rhythm across the AD continuum and is proportional to the progression of AD (Gouw et al., 2017; Cassani et al., 2018). Decreasing high frequency band power (i.e., alpha and beta), increasing low frequency band power (i.e., delta and theta), increasing the ratio between lower and higher frequency (i.e., theta-to-alpha ratio), and shifting of median frequency toward the lower band are quantitative manifestations of EEG slowing (Gouw et al., 2017; Cassani et al., 2018). Nevertheless, EEG slowing patterns were mostly reported in the dementia stages of AD rather than in the preclinical AD process. A few studies have suggested that although relative alpha power starts to decrease from the preclinical AD stage, alpha peak power does not shift until the prodromal stage of AD (López-Sanz et al., 2016). From the prodromal AD stage, these slower brain oscillations continuously increase along with the severity of cognitive impairment on the AD spectrum (Jafari et al., 2020). Notably, the brain’s slowing pattern might be also influenced by the existence of neurodegeneration and the individuals’ particular demographic characteristics (Gaubert et al., 2019; Babiloni et al., 2020). For example, the EEG slowing pattern has emerged in MCI (locally at temporal areas) and AD persons such that the power of low frequencies increases (Meghdadi et al., 2021), but in preclinical AD individuals with neurodegeneration, a decrease in low frequency oscillations is exhibited compared with those without neurodegeneration (Gaubert et al., 2019). People who exhibit subjective memory complaints and have a higher educational level might also have higher alpha power than those with lower educational levels (Babiloni et al., 2020).

Similarly, ERP latency has been used to reflect the temporal dynamics of brain activity in cognitively impaired individuals. In people with AD pathology, prolonged ERP latencies have been reported as a useful indicator for monitoring or even predicting the progression of age-related cognitive decline (Paitel et al., 2021).

In regards to cortical synchronization, the decrease in brain synchrony and complexity is likely due to reductions in cortical connections and neural network communication (Sun et al., 2020; Ranasinghe et al., 2021). A recent study demonstrated a significant decrease in functional connectivity in individuals with preclinical stages of AD within the medial parietal lobe (precuneus area particularly) in the default mode network using magnetoencephalography (Nakamura et al., 2018). The synchronization indexed by Granger causality matrices and local and global network connecting efficiency were also reduced in prodromal AD compared to the controls (Franciotti et al., 2019). On the other hand, subjective cognitive decline in elderly people with neurodegeneration demonstrated higher spectral entropy and complexity than those without neurodegeneration (Gaubert et al., 2019). These few studies on the preclinical and prodromal stages of AD suggest that EEG slowing, brain synchronization, and ERP delay in these very early stages of the AD continuum might still not be well understood. Therefore, more studies are needed to elucidate changes in EEG features, especially those that are correlated with amyloid beta deposition and neurodegeneration status.

Notably, these abovementioned findings were mostly manifested by EEG signals obtained from multichannel devices. However, with recent advancements in hardware and signal processing, few-channel EEG devices have increasingly been employed to examine neuroelectrical signals in research (Choi et al., 2019, 2020; Yi et al., 2019; Doan et al., 2021). Compared to multichannel EEG techniques, the simplicity of few-channel EEG systems can improve their practicality and portability while maintaining data quality (Gargiulo et al., 2010; Rogers et al., 2016). For example, a two-channel system such as prefrontal EEG techniques. Although the validity and reliability of prefrontal EEG in detecting cognitive impairment due to AD have been confirmed (Choi et al., 2019, 2020; Yi et al., 2019, 2021; Park et al., 2020), more studies employing this technique in the very early stages of the AD continuum are needed to rapidly enhance its practicality in clinical environments.

However, few-channel EEG studies on the early stages of AD that consider neurodegeneration information in addition to Aβ are scarce. Therefore, this study aimed to investigate whether the impact of amyloid load and neurodegeneration could be explained by prefrontal EEG and ERP markers. To evaluate the changes in prefrontal EEG/ERP metrics, we divided the subjects into four groups depending on their amyloid status and neurodegeneration status. The four groups were (1) prodromal AD (pAD) individuals who were amyloid positive and neurodegeneration positive (A+N+), corresponding to the MCI stage due to AD; (2) asymptomatic AD (aAD) individuals who were amyloid positive and neurodegeneration negative (A+N−); (3) MCI(-) individuals who were amyloid negative and neurodegeneration positive (A−N+), corresponding to “suspected non-Alzheimer’s pathologic change with MCI; and (4) cognitive normal (CN) individuals who were amyloid negative and neurodegeneration negative (A−N−). Amyloid-positive groups, pAD and aAD, belong to the Alzheimer’s disease continuum. We hypothesized that both amyloid-positive individuals would present specific EEG/ERP differences from CN or MCI subjects.

## 2. Materials and methods

### 2.1. Participants

We recruited one hundred seniors who were registered at the Gwangju Alzheimer’s Disease and Related Dementia (GARD) cohort to participate in this study. The participants aged between 66 and 83 years. The GARD database (Seo et al., 2016, 2018) includes participants who underwent several clinical examinations to determine their medical history, current medical status, demographic information, and cognitive ability. Structural and functional MRI (see Seo et al., 2016 for details) as well amyloid PET were performed to assess AD pathological aspects such as Aβ and neurodegeneration (Jack et al., 2018). Clinical Dementia Rating (CDR) was used to evaluate each participant’s cognitive function.

Cognitively unimpaired people were identified clinically as those who had a CDR score of zero and no sign of cognitive impairment; those with a CDR score of 0.5 and evidence of cognitive decline in one or more domains were considered MCI (Albert et al., 2011). Additionally, the presence of Aβ and neurodegeneration confirmed by PET scan and MRI results were used to further classify the participants into four subgroups, namely, the control group (CN, A−N−), asymptomatic with AD pathology (aAD, A+N−), MCI without AD pathology [MCI(−), A−N+], and MCI with AD pathology (pAD, A+N+) groups. The clinical diagnoses of probable AD status were designated according to the National Institute of Neurological and Communicative Disorders and Stroke–Alzheimer Disease and Research Disorders Association (NINCDS-ADRDA) criteria (McKhann et al., 2011).

All participants were tested with the hearing ability of rare tone (2,000 Hz) and standard tone (750 Hz) as well as the differences’ recognizing prior to the ERP tasks under the same auditory condition with the experiment (through earphones at the volume level of 70 dB). Some patients were excluded based on criteria detailed in Seo et al. (2016); (1) evidence of focal brain lesions on MRI including lacunes and white matter hyperintensity lesions of grade 2 or more according to the Fazekas scale (Fazekas et al., 1987), (2) Illiteracy, (3) severe visual or hearing loss, (4) any type of dementia, (5) any significant neurologic, medical, or psychiatric disorders which could affect mental function, and (6) current use of psychoactive medications. Consent was obtained from patients or their caregivers after providing them with complete descriptions of the purpose of the study. The study protocol was approved by the Institutional Review Board of Chonnam University Hospital (CNUH-2019-279) and was performed in accordance with the Declaration of Helsinki.

### 2.2. PET acquisition and processing

Accumulation of Aβ in the brain was measured with the ST PET-CT scanner (General Electric Medical Systems, Milwaukee, WI, USA). The participants underwent PET scanning for 90–100 min after the intravenous invasive injection of 20% ^18^F-florbetaben (NeuraCeq) with a mean dose of 303 MBq. The preprocessing of acquired PET images followed a previously described method (Choi et al., 2019). Trained specialists scored PET images based on the brain amyloid plaque load (BAPL) scoring system. PET scans with BAPL scores less than 2 were classified as Aβ-negative scans, and those with scores equal to or higher than 2 were considered Aβ-positive scans. The additional verification of amyloid positivity was performed by measuring the standardized uptake value ratio (SUVR) and considering SUVR values larger than 1.11 to be amyloid positive. All participants had no discrepancies between BAPL- and SUVR-based positivity assignments.

### 2.3. EEG/ERP acquisition and processing

All patients participated in EEG recordings in an upright seated position. There were two measurements taken: (1) an eye-closed resting-state recording in a quiet environment for 5 min and (2) a simple ERP measurement using a repetitive auditory pure tone as the stimulus for 8 min. Non-invasive monopolar scalp electrodes recorded electrical brain activity at two prefrontal regions (Fp1 and Fp2 in the International 10/20 electrode system) with a reference on the right earlobe. The neuroNicle amplifiers (LAXTHA Inc., Korea) had a bandpass filter from 3 to 43 Hz, and an input range of ±393 μV (input noise <0.6 μVrms). All filters were digital, and IIR Butterworth filters were applied as follows: (1) a 2nd order band stop filter with f_1_ = 55 Hz and f_2_ = 65 Hz, (2) a 1st order high-pass filter with f_*c*_ = 2.6 Hz, and (3) an 8th order low-pass filter with f_*c*_ = 43 Hz. The contact impedances were kept below 10 kΩ for each electrode. All data were digitized in continuous recording mode (5 min of EEG; 250 Hz sampling rate; 15-bit resolution).

To minimize ocular, muscular, and other types of artifacts in the resting-state recording, an operator monitored the subject and EEG traces, instructed the subject to remain in an eyes-closed and muscle-relaxed state, and alerted the subject whenever he or she showed signs of behavioral or EEG drowsiness. As we did not reject any artifacts during signal processing, we additionally tested for data contamination due to muscle and eye movement of the (Fp1, Fp2) prefrontal EEG. First, we checked that the EEG data were not contaminated by substantial artifacts for any subject. Specifically, none of the participants’ recordings contained more than 10% of epochs that exceeded a 200 μV maximum amplitude; this value is a common exclusion threshold for identifying serious artifacts (Noh et al., 2006). When applying a stricter voltage threshold, we still found no subjects with 10% of epochs exceeding 100 μV. Thus, none of the resting-state eyes-closed EEG data were rejected for artifacts in this study.

In the ERP protocol, stimuli comprised eight pure tones (125, 250, 500, 750, 1,500, 2,000, 3,000, and 4,000 Hz) that were played with equal probabilities. Tones were presented in pseudorandom order so that the tones of the same frequency were never presented consecutively. The participants were instructed to listen to them passively. A total of 480 stimuli were presented binaurally through insert earphones at a 70-dB SPL volume. The tone duration for each stimulus was 50 ms with rise and fall times of 1 ms. The interstimulus interval was 1 s. During the test, the subjects sat comfortably in a chair in an office room under regular lighting conditions.

### 2.4. EEG/ERP variables and computation

The present study focused on the following three representative EEG/ERP metrics related to AD: median frequency (MDF) and spectrum triangular index (STI) of resting-state EEG activity and positive peak latency (PPL) of ERPs. Reductions in MDF and STI reflect a slowing of brain oscillations and a decrease in coherence (or synchrony), respectively, whereas increased PPL suggests a delay in stimulus processing that occurs approximately 200 ms after the stimulus onset (canonically, P200).

Eye-closed resting-state EEG features are known to show intrinsic oscillation patterns that are dominant across the cortices (Choi et al., 2020). The EEG spectral distribution is approximately bell-shaped with a peak occurring in the 5–12 Hz region, which includes both theta (4–8 Hz) and alpha (8–13 Hz) bands. Moreover, in elderly individuals, the central frequency of this distribution is relatively slower than that of younger individuals, so the contribution of rhythm corresponding to the theta and alpha boundaries is quite high. Therefore, conventional EEG indices based on theta and alpha power are not suitable for quantifying the subtle slowing of intrinsic oscillation because the analysis is not sensitive to individual differences within standard frequency bands. Meanwhile, a peak frequency (PF) indicator, which is determined by the frequency of the highest peak point in the power spectrum, has been used in some studies. However, it is difficult to obtain consistent values in spectra with double peaks or side-spreading peaks. In the authors’ recent studies, the introduction of the median frequency (MDF) corresponding to half the area in a power spectral distribution (PSD) has yielded more significant features than other existing markers, such as the alpha-to-theta ratio (ATR) or peak frequency (PF) (Choi et al., 2019, 2020; Park et al., 2020; Doan et al., 2021). For these reasons, MDF was selected as a representative indicator for quantifying EEG slowing related to amyloid load in this study.

Two EEG variables were derived from a frequency-domain analysis of EEG data measured for 5 min; the frequency-domain (or spectral-domain) features are usually used in the quantitative analysis of EEG rhythms. To transform the EEG signal from the time-domain to the frequency-domain, a Fourier transform of the autocorrelation function was employed to calculate the power spectral density. The MDF measures the median frequency in the dominant intrinsic oscillatory frequency domain, and the STI measures the geometric triangular index of the spectral distribution.

The ERP marker, PPL, was extracted by the conventional ensemble averaging method to acquire an EEG response from auditory ERPs; it measures the time point corresponding to the maximum amplitude and is calculated relative to stimulus onset. All three variables were averaged over the left and right signals.

Concretely, the EEG power spectrum was obtained by fast Fourier transform (FFT) of the EEG signal with a rectangular window. The MDF was calculated in two steps. (1) All the spectral power values in the 5.5–13 Hz frequency domain were summed and divided by two. (2) The frequency was selected at which the cumulative power in the 5.5–13 Hz band first exceeded the value calculated in step (1). The STI was obtained as follows. (1) Total power was calculated as the sum of the spectral power values in the frequency range from 5.5 to 13 Hz. (2) Peak power was obtained by determining the maximum spectral power values in the same frequency range. (3) Peak/total percentage was defined simply as peak power divided by total power and multiplied by 100. (4) Finally, the ratio was converted to a natural logarithmic scale to fulfill the normal distributional assumption required for parametric statistical analysis (David et al., 2006).

The reliability of prefrontal EEG variables was verified in an authors’ previous study (Choi et al., 2020), which implies that the variables related to intrinsic EEG oscillation are interchangeable between prefrontal and other regions and behave as statistically stationary variables. The raw traces of four individuals representing each cognitive group with the measurements of selected EEG/ERP markers were illustrated in Supplementary Figure 1.

### 2.5. Statistical analysis

Demographic characteristics of each group were summarized by their means and standard deviations (SD) for quantitative variables and by their frequencies and percentages for qualitative variables. The significance level for all statistical tests was set to 0.05. All analyses were conducted using R (version 4.2.1, released 2022-06-23). To investigate the mean difference between CN and the other cognitively impaired states, generalized linear models (GLM) with the identity link function were fit to each EEG/ERP marker and controlled for cognitive-specific covariates such as age, sex, and education years. Since the cognitive states of aAD and pAD can be regarded as a progressive continuum from CN to AD, we conducted proportional odds ordinal logistic regression to identify the association of the sequential cognitive degeneration state [except for the MCI(-) group] with each EEG/ERP marker. All EEG/ERP variables satisfied the proportional odds assumption based on Brant tests (Brant, 1990). In addition, binary logistic regression analyses for CN vs. MCI(-), MCI(-) vs. aAD, and MCI(-) vs. pAD were conducted to investigate the possibility of EEG/ERP variables being able to discriminate MCI(-) from the AD continuum.

## 3. Results

### 3.1. Demographic information

The overall demographic information of the participants is summarized in Table 1. There were no significant differences in the major demographic features such as age, education years, and sex between the different cognitive groups (*p* > 0.05). However, the Mini-Mental State Examination (MMSE) total score significantly decreased across the AD spectrum (*p* = 0.017). There were no differences between the groups’ anthropometric measurements, blood pressure, daily habits, and comorbidities.

**TABLE 1:** Demographic characteristics, neuropsychological test, and related information.

Variables	CN (*n* = 27)^a^	MCI(-) (*n* = 42)^a^	aAD (*n* = 15)^a^	pAD (*n* = 16)^a^	*p*-Value^b^
Age (years)	73 (5)	74 (7)	77 (4)	76 (5)	0.082
Education year (years)	12.3 (4.3)	12.9 (4.1)	19.1 (22.5)	12.8 (5.7)	0.128
Female	13 (48%)	18 (43%)	12 (80%)	6 (38%)	0.242
MMSE	27.6 (1.8)	25.7 (2.7)	26.9 (2.3)	25.6 (3.4)	**0.017**
Height (cm)	158 (10)	158 (7)	161 (7)	160 (8)	0.516
Weight (kg)	63 (9)	60 (8)	66 (10)	62 (10)	0.264
Systolic BP (mmHg)	117 (11)	122 (17)	121 (10)	127 (21)	0.205
Diastolic BP (mmHg)	68 (9)	70 (12)	68 (6)	72 (12)	0.745
Alcohol consumption	1 (3.7%)	8 (19%)	2 (13%)	2 (12%)	0.317
Smoking habit	0 (0%)	1 (2.4%)	0 (0%)	0 (0%)	>0.999
Family history: dementia	5 (19%)	10 (24%)	4 (27%)	7 (44%)	0.640
Family history: stroke	3 (11%)	14 (33%)	5 (33%)	4 (25%)	0.173

BP, blood pressure; MMSE, Mini-Mental State Examination; CN, cognitive normal; MCI(-), mild cognitive impairment with amyloid negative and neurodegeneration positive; aAD, asymptomatic Alzheimer disease; pAD, mild cognitive impairment with Alzheimer’s pathology.

^a^*n* (%); Mean (SD).

^b^Oneway Analysis of Variance (ANOVA); Pearson’s Chi-squared test; Fisher’s exact test.

The *p*-value of lower than 0.05 were presented in boldface.

### 3.2. Association between EEG/ERP variables and cognitive states

Table 2 shows the mean differences and 95% confidence intervals (CI) of STI, MDF, and PPL variables between each of the cognitive impairment groups and the CN individuals. As a result, the pAD group had a significantly lower MDF and higher PPL than the CN group. These differences remained significant after controlling for age, sex, and education level and had mean differences (95% CI) of −0.40 (−0.78, −0.02) and 18.68 (2.90, 34.47), respectively. There were no significant changes in the MDF and PPL variables of the aAD or MCI(-) groups with respect to the CN group. In terms of STI, the mean differences between groups were not statistically significant. The correlations between each of the EEG/ERP variables as well as with the MMSE score were shown in Supplementary Figure 2.

**TABLE 2:** Estimated means for each cognitive state group and their mean differences from the CN group.

Variables	Mean (95% CI)	Contrast	Unadjusted		Adjusted^a^	
			**Mean difference** **(95% CI)^b^**	***p*** **-Value^b^**	**Mean difference** **(95% CI)^b^**	***p*** **-Value^b^**
**STI (%)**						
CN	−1.46 (−1.64, −1.28)					
MCI(−)	−1.48 (−1.62, −1.34)	MCI(−) vs. NC	−0.02 (−0.30, 0.26)	0.987	−0.05 (−0.32, 0.23)	0.931
aAD	−1.73 (−1.97, −1.48)	aAD vs. NC	−0.27 (−0.63, 0.10)	0.202	−0.36 (−0.74, 0.02)	0.069
pAD	−1.59 (−1.82, −1.36)	pAD vs. NC	−0.13 (−0.49, 0.23)	0.687	−0.18 (−0.54, 0.18)	0.472
**MDF (Hz)**						
CN	8.93 (8.75, 9.12)					
MCI(−)	8.90 (8.75, 9.06)	MCI(−) vs. NC	−0.03 (−0.32, 0.26)	0.976	−0.01 (−0.30, 0.28)	0.997
aAD	8.81 (8.55, 9.06)	aAD vs. NC	−0.13 (−0.51, 0.25)	0.740	−0.03 (−0.43, 0.37)	0.988
pAD	8.49 (8.24, 8.74)	pAD vs. NC	**−0.44 (−0.82, −0.07)**	**0.015**	**−0.40 (−0.78, −0.02)**	**0.036**
**PPL (ms)**						
CN	265.63 (257.78, 273.48)					
MCI(−)	276.00 (269.70, 282.30)	MCI(−) vs. NC	10.37 (−1.79, 22.53)	0.113	9.69 (−2.46, 21.85)	0.149
aAD	274.13 (263.60, 284.67)	aAD vs. NC	8.50 (−7.37, 24.38)	0.436	6.13 (−10.55, 22.81)	0.689
pAD	286.50 (276.30, 296.70)	pAD vs. NC	**20.87 (5.31, 36.43)**	**0.005**	**18.68 (2.90, 34.47)**	**0.016**

Unadjusted or adjusted mean differences were derived from the GLM with identity link function. STI, spectrum triangular index; MDF, median frequency; PPL, positive peak latency; CN, cognitive normal; MCI, mild cognitive impairment with amyloid negative and neurodegeneration positive; aAD, asymptomatic Alzheimer disease; pAD, mild cognitive impairment with Alzheimer’s pathology.

^a^Adjusted for sex, age, and education years.

^b^95% confidence interval (CI) and *p*-values and were obtained from t statistics and were adjusted using Dunnett’s method.

The *p*-value of lower than 0.05 were presented in boldface.

### 3.3. Association between EEG/ERP variables and AD continuum

With the exception of the MCI(-) group, we investigated the association between the EEG/ERP variables and the cognitive groups using proportional ordinal logistic regression models. The model results are presented in Table 3. The MDF and PPL variables were consistently significantly associated with the aAD and pAD stages after adjusting for age, sex, and education levels with odds ratios (95% CI) of 0.58 (0.34,0.97) and 2.36 (1.31, 4.78), respectively. STI did not show a significant association with the occurrence of aAD and pAD.

**TABLE 3:** Association of the EEG and ERP variables between AD continuum states of CN, aAD and pAD.

Ref: CN group Variables	Unadjusted		Adjusted^b^	
	**Odds ratio^a^** **(95% CI)**	**Z; *p*-value**	**Odds ratio** **(95% CI)**	**Z; *p*-value**
STI (%)	0.74 (0.44, 1.21)	−1.18; 0.237	0.69 (0.40, 1.15)	−1.38; 0.167
MDF (Hz)	**0.54 (0.32, 0.87)**	**−2.45; 0.014**	**0.58 (0.34, 0.97)**	**−2.01; 0.044**
PPL (ms)	**2.35 (1.37, 4.40)**	**2.92; 0.004**	**2.36 (1.31, 4.78)**	**2.65; 0.008**

STI, spectrum triangular index; MDF, median frequency; PPL, positive peak latency; CN, cognitive normal; aAD, asymptomatic Alzheimer disease; pAD, mild cognitive impairment with Alzheimer’s pathology.

^a^Estimated using proportional odds ordinal logistic regression analysis.

^b^Adjusted for sex, age, and education.

The *p*-value of lower than 0.05 were presented in boldface.

### 3.4. EEG/ERP variables in MCI(-) compared with the AD pathological stages

Finally, we investigated associations of the three EEG/ERP variables between MCI(-) and the other cognitive groups using binary logistic regression models, as shown in Table 4. In comparison to the CN group, the MCI(-) people exhibited significant prolonged PPL. A one-unit increase in PPL corresponded to an increase of 85% the odds of having MCI(-). With respect to the MCI(-) group, MDF and STI demonstrated negative associations with the aAD and pAD groups. In particular, a one-unit decrease in STI corresponded to an increase of approximately 70% the odds of having aAD, and a one-unit decrease in MDF corresponded to an increase of approximately 58% in the odds of having pAD. These findings suggested that MCI(-) individuals had a longer PPL than those with CN and greater STI and higher MDF than those with aAD and pAD, respectively.

**TABLE 4:** Association of EEG and ERP variables between MCI(-) and the other cognitive states.

	MCI(-) vs. CN				aAD vs. MCI(-)				pAD vs. MCI(-)			
	**Unadjusted**		**Adjusted** **^b^**		**Unadjusted**		**Adjusted** **^b^**		**Unadjusted**		**Adjusted** **^b^**	
	**Odds ratio (95% CI)^a^**	**Z; *p*-value**	**Odds ratio (95% CI)^a^**	**Z; *p*-value**	**Odds ratio (95% CI)^a^**	**Z; *p*-value**	**Odds ratio (95% CI)^a^**	**Z; *p*-value**	**Odds ratio (95% CI)^a^**	**Z; *p*-value**	**Odds ratio (95% CI)^a^**	**Z; *p*-value**
STI [%]	0.96 (0.59, 1.57)	−0.17; 0.862	0.90 (0.54, 1.51)	−0.40; 0.688	0.48 (0.21, 0.95)	−1.95; 0.052	**0.30** **(0.10, 0.74)**	**−2.33;** **0.020**	0.77 (0.41, 1.39)	−0.84; 0.403	0.73 (0.37, 1.35)	−0.97; 0.330
MDF [Hz]	0.94 (0.57, 1.53)	−0.27; 0.788	0.95 (0.58, 1.57)	−0.19; 0.853	0.81 (0.44, 1.48)	−0.67; 0.502	0.96 (0.48, 1.94)	−0.11; 0.910	**0.41** **(0.19, 0.78)**	**−2.54;** **0.011**	**0.42** **(0.19, 0.83)**	**−2.32;** **0.021**
PPL [ms]	**1.85** **(1.08, 3.48)**	**2.07;** **0.038**	**1.82** **(1.04, 3.50)**	**1.96;** **0.050**	0.91 (0.48, 1.64)	−0.31; 0.758	0.76 (0.36, 1.49)	−0.76; 0.446	1.55 (0.88, 2.82)	1.51; 0.132	1.47 (0.80, 2.75)	1.24; 0.214

^a^Unadjusted and adjusted odds ratio and their 95% confidence intervals (CI) were obtained from results of the binary logistic regression analysis.

^b^Adjusted for sex, age, and education.

STI, spectrum triangular index; MDF, median frequency; PPL, positive peak latency; CN, cognitive normal; MCI(-), mild cognitive impairment with amyloid negative and neurodegeneration positive; aAD, asymptomatic Alzheimer disease; pAD, mild cognitive impairment with Alzheimer’s pathology. The *p*-value of lower than 0.05 were presented in boldface.

## 4. Discussion

This study revealed relationships between prefrontal resting-state EEG/ERP variables in the very early stages of AD pathology as well as in non-AD MCI conditions. Overall, we found that the pAD group exhibited a shift of MDF toward a lower frequency and a delay in the PPL compared to the CN group (Tables 2, 3). Although the *p*-value did not reach the significance threshold, individuals with aAD also exhibited a trend of reduced brain synchrony indicated by a decrease in STI compared with CN individuals (Table 2). Additionally, the non-AD MCI group demonstrated a pattern of longer PPLs with respect to the CN group, faster EEG rhythms than the pAD group, and lower synchronization than the aAD individuals. These results were found after controlling for potential confounders such as age, sex, and educational level.

Previous studies have reported significant changes in EEG/ERP features in individuals with cognitive impairment due to AD. Slower EEG rhythms, a loss of signal synchronization, and a delay in stimulus- response time are commonly found, especially in stages of the AD spectrum that are associated with dementia (Jeong, 2004; Dauwels et al., 2011; Howe et al., 2014; Fruehwirt et al., 2019). Our findings in these features are consistent with these studies. Nevertheless, these patterns were not homogeneously exhibited in the preclinical AD stage and people with non-AD MCI (Gaubert et al., 2019; Meghdadi et al., 2021).

First of all, in terms of MDF, we found that the pAD group exhibited significant differences with the CN individuals, while MCI(-) had no significant relationship with MDF when compared with the CN individuals. Additionally, although the aAD group showed a significant association with MDF in Table 3, MDF did not significantly differ between aAD and CN as shown in Table 2. These results suggested that slowing of brain oscillations which was indicated by a reduction of MDF did not occur in the aAD stage or MCI(-) but was only present in a later stage of pAD when neurodegeneration appeared. Meghdadi et al. (2021) reported a similar slowing in EEG oscillation in the AD group but not strongly significant in the MCI individuals by an increase of spectral power at the slower frequencies (Delta, Theta) on a twenty-channel EEG device. The other studies also reported slower EEG patterns in MCI and AD groups from the multi-channel EEG techniques (Czigler et al., 2008; Ya et al., 2015). The slowing of brain oscillations is thought to be caused by the loss of cholinergic innervation, which is indicated by the reductions in cholinergic-monoaminergic interactions and atrophy of cholinergic nuclei (Dringenberg, 2000; Schumacher et al., 2020). This cholinergic denervation mechanism remains active in the early stages, such as MCI (Mesulam et al., 2004; Teipel et al., 2011), and even before the onset of clinical symptoms due to AD (Beach et al., 2000). Nonetheless, this suggested that EEG slowing may be an AD-specific pattern and can only be observed from the pAD stage of the continuum.

Previous studies have demonstrated a decrease in prefrontal MDF in eyes-closed resting-state EEG in cognitively healthy participants (Choi et al., 2020) that positively correlated with the MMSE score (Choi et al., 2019). In this study, MDF exhibited a significant positive correlation with the MMSE score upon controlling for the potential covariates such as age, sex, and education years as shown in Supplementary Figure 2. In addition, this MDF reduction has been found in patients experiencing a decline of brain functions, such as individuals with postoperative delirium or those exposed to methyl bromide. For instance, the postoperative delirium group showed a lower preoperative MDF than the non-delirium group; surprisingly, a sharp decrease in prefrontal MDF was observed after surgery under general anesthesia in both groups, and this effect remained even after 1 month (Kim et al., 2022). Similarly, a significant decrease was observed in prefrontal MDF after work exposure to methyl bromide, which is a fumigant that is widely used for killing pests (Park et al., 2020). These previous studies consistently pointed out that prefrontal MDF reduction is related to a decline in brain functions. Similar to anesthetics and pesticides, amyloid plaque deposition is neurotoxic, which may cause MDF lowering in the group with a high amyloid load.

On the other hand, we found that the aAD group, who had Aβ deposition but had not yet shown neurodegeneration damage, showed a trend of a reduction in EEG signal synchronization compared to the CN (Table 2) and MCI(-) persons (Table 4). Relationships between neuro-desynchronization and the reduction in neuronal communication and cortical connections have also been previously reported (Sun et al., 2020; Ranasinghe et al., 2021). However, this reduction did not remain significant in pAD individuals, as shown by insignificant ORs and mean differences from the CN and MCI groups (Tables 2, 4). A recent study showed that functional connectivity across long-term neural networks decreases (becoming more abnormal) with the progression of AD neurodegeneration (Bokde et al., 2009). If the functional connectivity in the resting state network is strong, synchronization with enhanced temporal phase modulation occurs, which induces an increase in spectral power of EEG activity around the synchronized frequency and consequently increases the STI value. In this study, the aAD group showed a reduced STI compared to the non-AD MCI patients, and this association remained significant after adjusting for age, sex, and education levels, as shown in Table 4. A previous study reported that patients with mild AD featured a prominent reduction in the likelihood of synchronization of alpha rhythms in fronto-parietal regions compared to non-Alzheimer’s dementia patients (vascular dementia). They suggested that mild AD patients were characterized by the fronto-parietal coupling of dominant alpha rhythms (Babiloni et al., 2004). This report is in alignment with the STI results in this study in the aAD group when compared with the MCI(-) individuals. Although the aAD group exhibited a lower mean STI than the CN group, the difference was not statistically significant which might be due to the small sample size. Therefore, future studies with larger sample sizes are needed to understand the change in EEG synchrony in the early stages of the AD continuum.

Furthermore, the PPL was found to be related to pAD and MCI(-). We found a significantly prolonged PPL in pAD and MCI(-) patients, as indicated by higher mean values and odds ratios that were greater than one. The prolonged peak latency was suggested to be caused by a reduction in cognitive processing ability requiring participants to need more time to process sensory information. Nevertheless, this result suggested that this delay in the sensory function of the brain might not represent AD-specific changes but rather a general cognitive decline. ERP responses to auditory stimuli contain discriminative information that predicts which MCI patients are likely to progress to more severe cognitive impairment (Bennys et al., 2011), and ERP variables of cognitive function are increasingly affected in longitudinal studies on MCI and AD patients (Lai et al., 2010; Papaliagkas et al., 2011). Moreover, people with the causative genes of AD such as amyloid precursor protein (APP) and presenilin 1 (PSEN1) showed significant ERP changes even before the onset of AD-related behavioral abnormalities (Golob et al., 2009; Quiroz et al., 2011). Golob et al.’s (2009) showed significantly delayed latencies for the early ERP components among familial AD mutation carriers. In an ERP, the P200 peak that occurs at approximately two hundred milliseconds after stimulus onset is considered to reflect exogenous sensory attention (Katada et al., 2004). Delayed P200 latency has been found in patients with sporadic or familial AD and other abnormalities (Martinelli et al., 1996; Missonnier et al., 2007), indicating that early sensory-cognitive processes might be compromised in AD. We were also able to confirm that the PPL marker, corresponding to the latency of P200 in auditory ERP, became delayed with AD progression, as shown in Tables 2, 3. This is consistent with our previous result; PPL showed a negative correlation with the MMSE score (Supplementary Figure 2; Doan et al., 2021) and was delayed in the dementia group compared to the non-dementia group (Doan et al., 2021).

There have been recent studies on the predictive utility of EEG/ERP variables for dementia. Compared with other imaging modalities, EEG/ERP can promptly detect subtle changes in brain function, especially those indicating early-stage cognitive decline, due to its high temporal resolution. While most previous studies used multichannel EEG techniques, this study used a simple and wearable EEG band with two prefrontal channels at Fp1 and Fp2. A previous study reported that prefrontal EEG/ERP variables outperformed MMSE scores in predicting dementia; combining both measures can increase prediction accuracy (Doan et al., 2021). Therefore, prefrontal EEG/ERP has wide potential applications as a non-invasive, wearable, and cost-effective screening tool for cognitive impairment. To the best of our knowledge, however, there have been no studies on the predictive utility of prefrontal EEG/ERP variables in the very early stages of the AD continuum. Although data analysis from EEG has already led to the proposal of putative biomarkers for MCI and AD, studies on cognitively normal Alzheimer’s or non-Alzheimer’s MCI were rare. Our study is the first report on the EEG/ERP variable changes in the preclinical and prodromal stages due to AD.

The EEG variables of MDF and STI and the ERP variable of PPL used in the current study indicate the functional degeneration corresponding to the functional slowing and reduced connection in the resting state network (RSN) and task positive network (TPN), respectively. Those variables have low dependence on absolute signal amplitude, thus not sensitive to scalp thickness or contact resistance, and low dependence on delta and gamma bands, thus not sensitive to movement or muscle contraction (Definitions and detailed calculation methods for MDF, STI, and PPL are described in detail in “section 2.4. EEG/ERP variables and computation,” in which low dependences on signal amplitude and on delta/gamma bands can be easily inferred). Therefore, MDF, STI, and PPL variables have high reproducibility and robustness against artifacts and scalp morphology variabilities between individuals, which is advantageous to practicality in real clinical usage.

Meanwhile, MDF decrease has been reported in various disease conditions such as aging, cognitive decline, toxic exposure, and postoperative delirium among others. It implies that MDF decrease and probably STI and PPL changes may not be AD-specific but reflects brain functional changes occurred by AD progression, which is a limitation of EEG variables in general.

Several limitations of this study need to be addressed. Mainly, the number of participants for each group was relatively small, thus limiting the uses of more advanced EEG/ERP features as well as the generalizations of these results. A larger cohort can help validate these findings as well as additionally elucidate the relationship between EEG/ERP variables and predementia stages of the AD spectrum. Additionally, we were not able to observe and monitor the participants over time. Longitudinal studies may help provide additional insight into how these EEG/ERP features change along with the progression of AD dementia.

## 5. Conclusion

This study employed two resting-state EEG variables and one sensory ERP marker obtained from the two prefrontal EEG channels to examine the associations with predementia stages of AD and non-AD MCI. We found that a slower intrinsic EEG oscillation was associated with MCI due to AD (prodromal AD), whereas a prolonged ERP latency was more likely to be related to global cognitive impairment rather than AD-specific impairment. Additionally, a trend of reduced brain synchronization was observed in the preclinical stage of AD. These findings provided additional insight into how EEG/ERP features change in the very early stages of the AD continuum and suggested that prefrontal EEG/ERP variables can serve as potential markers for screening early stages of cognitive impairment due to AD.

## Data availability statement

The datasets presented in this article are not readily available because of ethical and access restrictions. Requests to access the datasets should be directed to the corresponding authors.

## Ethics statement

The study protocol was approved by the Institutional Review Board of Chonnam University Hospital (CNUH-2019-279). The patients/participants provided their written informed consent to participate in this study.

## Author contributions

JC led the manuscript preparation. BK analyzed the data and wrote the manuscript. DD and JP handled the manuscript qualification and reviewed the manuscript. JC and WC extracted the relevant EEG/ERP variables and controlled the data quality. KL designed and led the project, obtained the institutional review board approval, and managed the data. JK supervised and wrote the manuscript. All authors revised and approved the contents of the manuscript, contributed to the article, and approved the submitted version.
