# Supplementary material for: Prefrontal EEG slowing, synchronization, and ERP peak latency in association with predementia stages of Alzheimer’s disease
Source: Front Aging Neurosci. 2023 Mar 23;15:1131857. doi: 10.3389/fnagi.2023.1131857 (PMC10076640; doi:10.3389/fnagi.2023.1131857)
Supplement: Supplementary file 1 [file Table_1.DOCX]

Supplementary Material

Prefrontal EEG slowing, synchronization, and ERP peak latency in association with predementia stages of Alzheimer’s disease

**Jungmi Choi^1^, Boncho Ku^2^, Dieu Ni Thi Doan^2,3^, Junwoo Park**^4^**, Wonseok Cha^1^,**

**Jaeuk U. Kim^2,3,*^, Kun Ho Lee^4,5,6,*^**

^1^Human Anti-Aging Standards Research Institute, Uiryeong-gun, Gyeongsangnam-do, South Korea

^2^Digital Health Research Division, Korea Institute of Oriental Medicine, Daejeon, South Korea

^3^School of Korean Convergence Medical Science, University of Science and Technology, Daejeon, South Korea

^4^Gwangju Alzheimer’s Disease and Related Dementias (GARD) Cohort Research Center, Chosun University, Gwangju, South Korea

^5^Department of Biomedical Science, Chosun University, Gwangju, South Korea

^6^Dementia Research Group, Korea Brain Research Institute, Daegu, South Korea

*** Correspondence:** Jaeuk U. Kim, *jaeukkim@kiom.re.kr;* Kun Ho Lee, *leekho@chosun.ac.kr*

# Supplementary Figures

# Figure S1. Illustrations of the EEG and ERP raw signals from four representatives of each cognitive group.

#
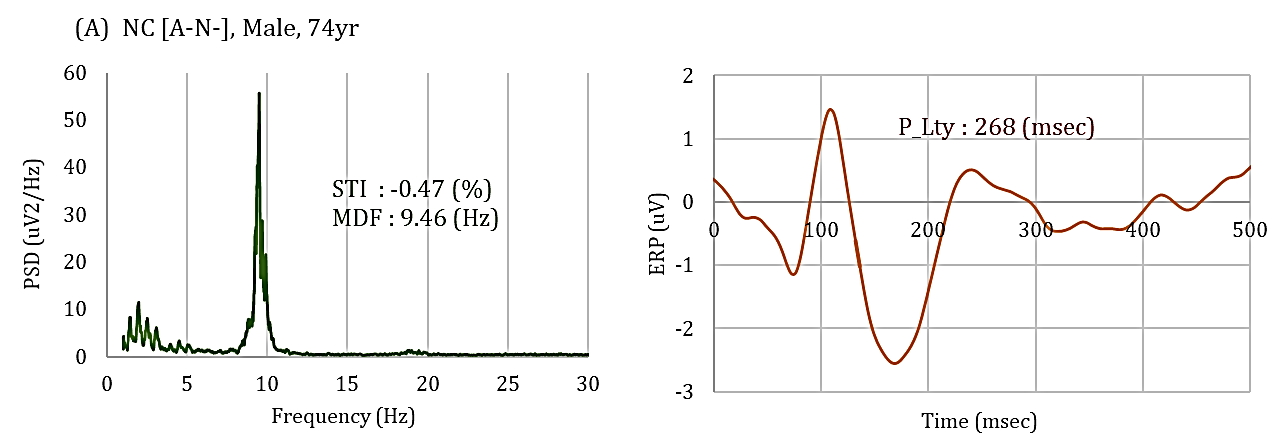

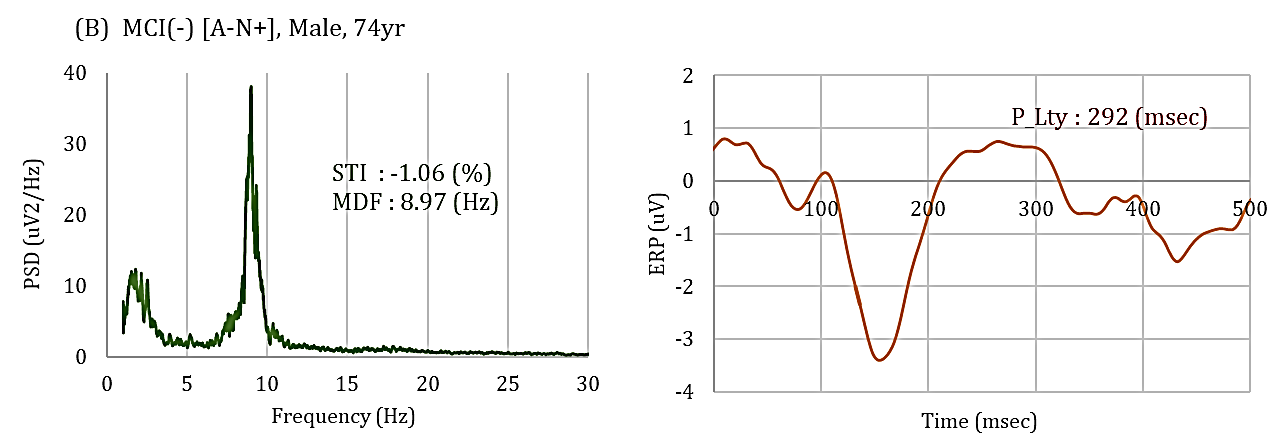

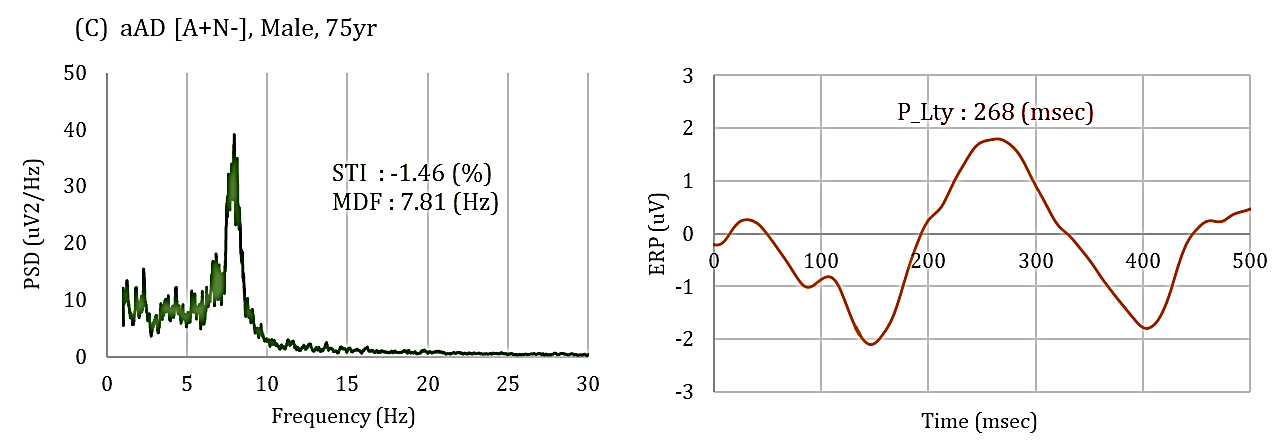

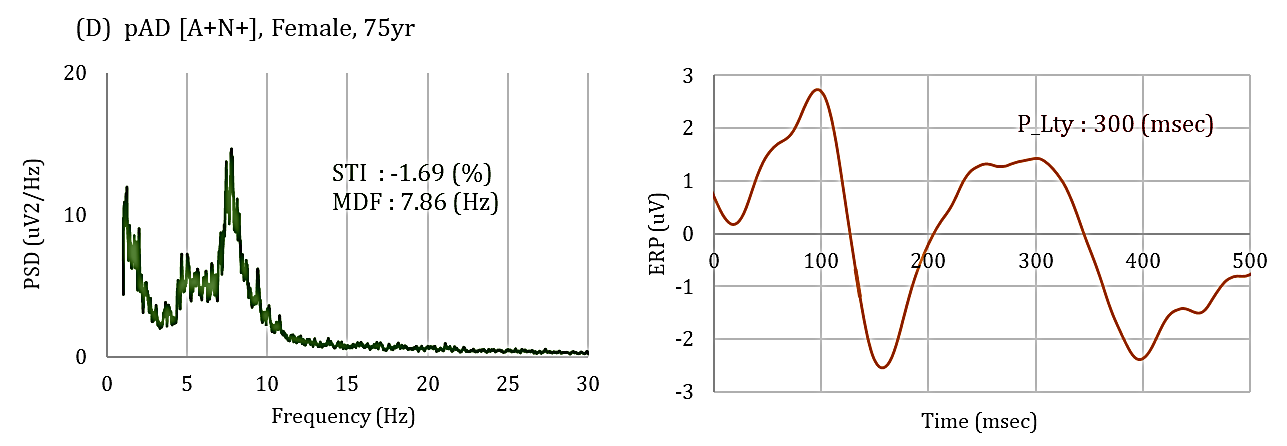


**Figure S1**. Illustrations of the EEG and ERP markers of four individuals representing each cognitive groups of NC, MCI(-), aAD, and pAD. The values of each EEG/ERP variables are displaying on the graphs. A shifting towards lower frequency of MDF, a reduced STI, and a prolonged PPL are shown along with the severity of cognitive stages.

# Figure S2. Partial correlation between K-MMSE score and the EEG/ERP variables.

**
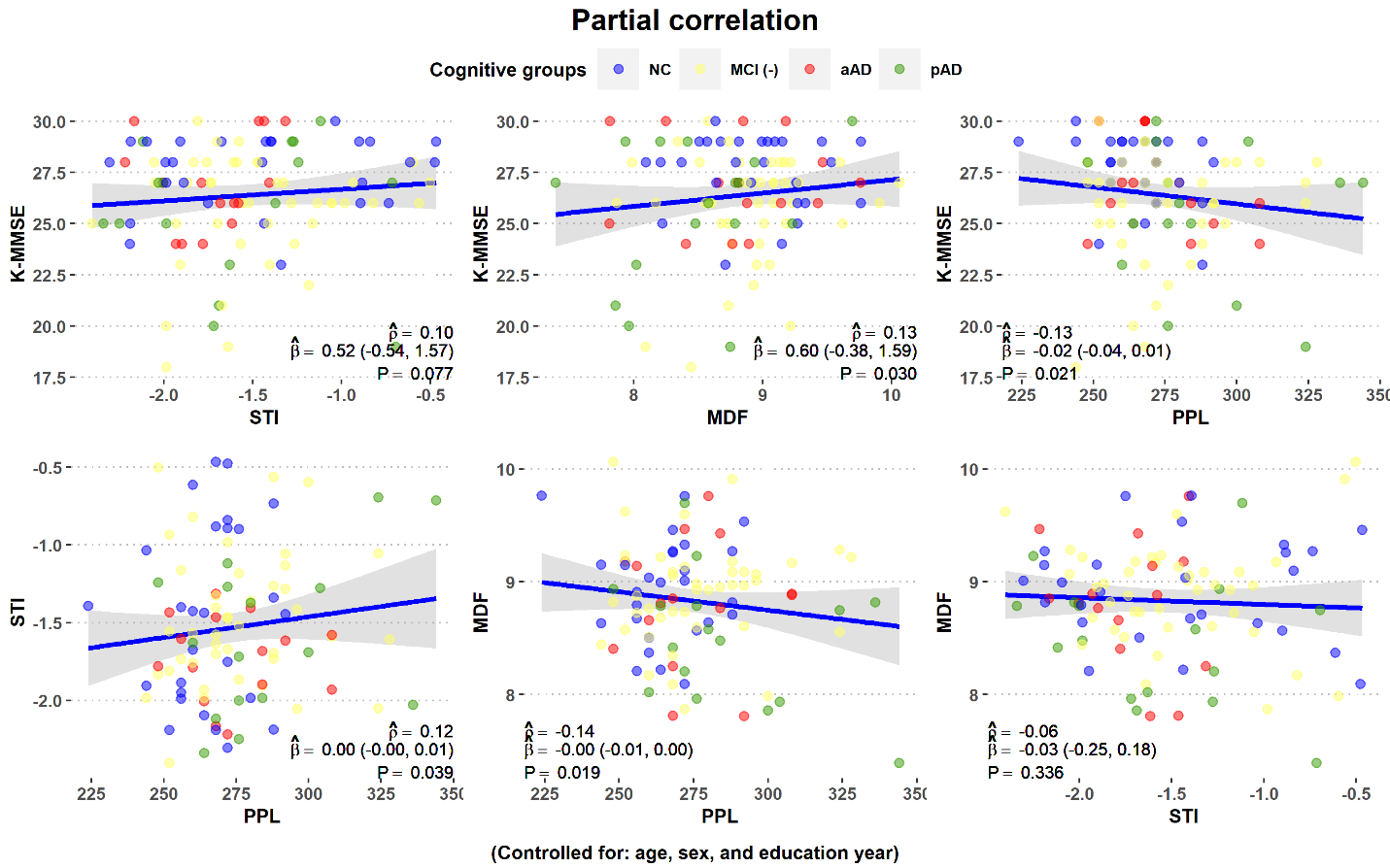
**

**Figure S2**. Scatterplots between MMSE scores, EEG, and ERP features. The circles indicate individual data coloring according to the cognitive groups. The blue line and shaded area show the estimated regression curves and 95 % CIs derived from the univariate regression analysis. The estimated values with their 95% CIs of Pearson’s correlation coefficients and regression coefficients for each EEG/ERP measures are shown from partial correlation upon controlling for age, sex, and education year.
